# Hypoalbuminemia after pancreaticoduodenectomy does not predict or affect short-term postoperative prognosis

**DOI:** 10.1186/s12893-020-00739-2

**Published:** 2020-04-15

**Authors:** Wei Xu, Xiangqun Peng, Bo Jiang

**Affiliations:** Department of Hepatobiliary Surgery, Hunan Provincial People’s Hospital, The First Hospital Affiliated with Hunan Normal University, No. 61 West Jiefang Road, Changsha, 410005 China

**Keywords:** Abdominal surgery, Outcomes, Complication, Pancreaticoduodenectomy, Hypoalbuminemia, Albumin

## Abstract

**Background:**

Hypoalbuminemia (HA) is a risk factor for the complications following pancreaticoduodenectomy (PD). This study aimed to explore the factors that affect HA following PD and evaluate the influence of HA on the short-term postoperative prognosis.

Methods: Total 163 patients who underwent PD and met inclusion criteria were subdivided into two groups according to the status of HA. The relationships of postoperative albumin (ALB) level and exogenous ALB infusion with postoperative responses and complications were assessed by correlation analysis.

**Results:**

Preoperative ALB ≥35.0 g/L and postoperative complication grade were factors influencing HA after PD. Correlation analysis demonstrated significant negative correlation of postoperative ALB level with white blood cell (WBC) count and neutrophil count. Postoperative exogenous ALB infusion positively correlated with blood urea nitrogen, creatinine, complication grade, postoperative intraperitoneal hemorrhage and pancreatic fistula. No significant differences were observed between the complications and30-day mortality rates with and without postoperative HA.

**Conclusions:**

HA after PD should not be considered as an indicator but rather a result of poor prognosis. WBCs, especially neutrophils, are involved in reducing postoperative ALB level. Infusion of exogenous ALB to maintain ALB > 30 g/L could not improve clinical outcomes.

## Background

Pancreaticoduodenectomy (PD) is a complicated abdominal surgical procedure and postoperative mortality for PD has decreased to 3–5% with improvements in the surgical technique and perioperative management [1–3]. However, the complication rate following PD is still as high as 30–60% [4, 5]. Hypoalbuminemia (HA) is a risk factor for pancreatic fistula and other complications such as bleeding, intra-abdominal abscess formation, and multiple organ failure, thereby increasing postoperative mortality rate [6, 7]. Multiple studies have reported that HA may increase the incidence of complications and mortality, prolong ICU admission and hospital stay, and increase the cost of medical resources [8, 9]. Infusion of exogenous albumin (ALB) was reported to improve clinical outcomes in several studies, although other studies reached the opposite conclusion [10–14]. Despite inconsistent conclusions, clinicians often aim to increase postoperative ALB level to a so-called “safe” range to improve patient condition. However, the cause and effect relationship between HA and postoperative prognosis is still widely disputed. As a challenging abdominal surgical procedure, PD comprises various types of abdominal surgeries including organ resection and digestive tract reconstruction. It is of great clinical significance to investigate the factors that influence postoperative HA and related complications in PD compared with other abdominal operations.

The postoperative ALB level is affected by the recovery process, complications, and daily fluid therapy management strategy, such as the ratio of crystalloid/colloidal fluid. Therefore, this study aimed to explore the factors influencing postoperative HA and assess the relationship between ALB changes and complications after PD. In addition, we aimed to investigate the role of exogenous albumin in the prognosis of patients after PD.

## Methods

### Patients

All subjects have given written informed consent and the study protocol was approved by Ethics Committee of Hunan Provincial People’s Hospital, The First Hospital Affiliated with Hunan Normal University (Approval No. 2702, Date 2015-12-04). In this prospective study, 238 consecutive patients who had undergone PD during two-year period between January 2016 and January 2018 in our hospital were initially enrolled. Patients were excluded if they underwent: (1) pancreatogastrostomy reconstruction; (2) Braun anastomosis in digestive tract reconstruction; and (3) total laparoscopic PD and conversion to open procedure, to avoid the interference of additional procedures on patient prognosis. All patients were subdivided into two groups according to the postoperative status of HA: HA group and non-HA group. HA was defined by serum ALB level ≤ 30 g/L until the 14th day after operation independent of the supplementation with exogenous ALB.

### Preoperative examination and radiographic assessment

Routine blood tests, biochemical examination, measurement of carbohydrate antigen 19–9 (CA19–9), abdominal ultrasound, computed tomography (CT), magnetic resonance imaging (MRI)/magnetic resonance cholangiopancreatography (MRCP), and endoscopic ultrasound (EUS) were performed before operation. Only preoperatively latest examination results were adopted in this study. Routine blood tests, liver and kidney function tests, electrolyte levels, coagulation, and C-reactive protein (CRP) level, amylase levels in blood and drainage, and lipase detection were examined on the 1, 3, 5, 7, 10 and 14 days after operation. Postoperative abdominal ultrasound and/or CT were applied to evaluate ascites routinely.

### Operative procedure

Classical PD was performed for all patients. Briefly, after kocherization of the pancreatic head and duodenum, the pancreas was divided anteriorly and to the left of the superior mesenteric vein and portal vein. En bloc removal of each specimen involved distal stomach resection at 10–40% and removal of all of the duodenum, gallbladder, and common bile duct (transected at the cystic duct confluence level) as well as approximately 10–20 cm of the proximal jejunum distal to the ligament of Treitz, with resection of the head, neck, and uncinate process of the pancreas. The scope of lymphadenectomy was dependent on the nature of the lesions and observations on operative exploration. Types of pancreaticojejunostomy reconstruction comprised of duct-to-mucosa end-to-side pancreaticojejunostomy with internal or external stent, invagination pancreaticojejunostomy with internal or external stent, or pancreaticojejunostomy without stent application. The stenting silicone tube could be guided externally through the jejunal loop (external stent) or cut short and left in the jejunal loop (internal stent).

### Definition and classification of complications

Postoperative complications were graded according to the Clavien–Dindo criteria [15]. Postoperative pancreatic fistula (POPF) was defined by an amylase concentration in the drainage fluid exceeding three times the upper limit of normal value or pancreatic intestinal anastomotic rupture on imaging [16]. Delayed gastric emptying (DGE) was defined by: 1) nasogastric tube decompression ≥3 days and meeting one of the following standards: a) vomiting after nasogastric tube removal, b) medication needed to improve gastrointestinal motility at 10 days postoperatively, c) replacement of the gastric tube, and d) inability to tolerate a solid diet by 7 days after operation; or 2) nasogastric tube decompression ≥10 days and meeting two of the explained standards [17]. Postpancreatectomy hemorrhage (PPH) cases were divided into early stage hemorrhage within 24 h after operation and late stage hemorrhage occurring more than 24 h after operation [18]. Ascites was detected by postoperative abdominal ultrasound or CT after exclusion of pancreatic fistula, biliary fistula, anastomotic fistula, or chylous ascites, and the ascites severity was further graded as mild when the depth of effusion was < 3 cm or the daily drainage was < 50 ml, as middle when the depth of effusion was 3–5 cm or the daily drainage was between 50 and 500 ml, and large when the depth of effusion was ≥5 cm or the daily drainage exceeded 500 ml. Hyperbilirubinemia was defined by blood TBIL ≥171.0 μmol/L, postoperative TBIL elevation to ≥171.0 μmol/L in patients with a preoperative TBIL < 171.0 μmol/L, or postoperative elevation to ≥171.0 μmol/L after transient decline. Infectious complications included incision infection, intra-abdominal abscess, anastomotic fistula, pneumonia, and septicemia [19].

### Postoperative management

All patients received standard care for postoperative management. Somatostatin (3 mg/Q12h) was administered to all patients by transfusion with a micro-infusion pump through postoperative day 7 or extended according to postoperative complications. A gastric tube was routinely placed preoperatively and removed after the recovery of gastrointestinal function. The drainage tube was removed if there was no evidence of biliary fistula or pancreatic fistula. Total parenteral nutrition was used for patients with an inadequate diet and stopped when the patients could tolerate at least half of their oral diet. An exogenous ALB preparation (Baxter Healthcare, Deerfield, USA) was adopted to maintain ALB level > 30 g/L. The endpoint was short-term analysis of early postoperative results.

### Statistical analysis

Measurement data are depicted as median (minimum - maximum). Rates were compared by Pearson *x*^*2*^ test, Fisher’s test, or Wilcoxon rank sum test. The medians were compared by the Mann–Whitney *U* test. Binary logistic regression was used for postoperative univariate and multivariate analyses. Canonical correlation analysis was used to evaluate the relationship of postoperative ALB level and infused amount with indicators and complications. A standardized canonical coefficient (SCC) > 0.5 was considered relevant. All statistical analysis was performed using PASW Statistics software 18.0 (SPSS Inc., Chicago, USA). *P* < 0.05 was considered significant.

## Results

### Patient characteristics

A total of 238 patients underwent PD between January 2016 and January 2018 in our hospital. Of these, 13 cases with pancreatogastrostomy reconstruction, 15 cases with Braun anastomosis in the digestive tract reconstruction, 43 cases with total laparoscopic PD, and 4 cases that required conversion to laparotomy were excluded. The remaining 163 patients were enrolled in the present study, including 35 cases of lower common bile duct cancer, 49 cases of duodenal papilla carcinoma, 49 cases of pancreatic head carcinoma, 17 cases of periampullary carcinoma, and 13 cases with an inflammatory mass at the head of the pancreas. On the postoperative pathology, 140 cases were confirmed to be adenocarcinoma, 2 cases were adenosquamous carcinoma, 5 cases were mucinous adenocarcinoma, 12 cases involved an inflammatory mass, 2 cases were cystadenocarcinoma, 1 case was sarcoma, and 1 case involved a retention cyst. The ratio of men to women was 1.3:1, and the average patient age was 57.8 years (range, 36–79 years; Table 1).

**Table 1:** Analysis of potential factors as predictors of postoperative HA

Factor	HA(*n* = 61)	non-HA(*n* = 102)	Univariate			Multivariate		
			OR	95% CI	*P value*	OR	95% CI	*P value*
***Patient characteristics***								
Gender								
Male	29	62	1					
Female	32	40	0.604	0.317–1.149	0.124			
Age (years)^a^	59.6 ± 8.5	56.6 ± 9.7	1.036	1.000–1.074	0.051			
Weight loss > 5 kg								
Yes	16	31	1					
No	45	71	0.621	0.284–1.357	0.232			
BMI(kg/m^2^)^a^	21.6 ± 3.1	21.6 ± 3.3	1.000	0.884–1.131	0.996			
Diabetes mellitus								
No	58	97	1					
Yes	3	5	1.021	0.235–4.433	0.978			
Acute pancreatitis								
No	59	98	1					
Yes	2	4	0.845	0.150–4.757	0.848			
Chronic pancreatitis								
No	42	57	1					
Yes	19	45	1.021	0.235–4.433	0.978			
Preoperative biliary drainage								
No	58	94	1					
Yes	3	8	0.618	0.158–2.427	0.491			
Preoperative ALB usage								
No	58	97	1					
Yes	3	5	0.276	0.046–1.638	0.157			
Preoperative ALB usage ^a^(g)	14.7 ± 4.2	34.5 ± 9.4	0.999	0.975–1.023	0.910			
WBC(× 10^9^/L)^a^	6.6 ± 2.3	6.3 ± 2.1	1.074	0.926–1.247	0.346			
NEUT(×10^9^/L)^a^	4.6 ± 2.0	4.3 ± 1.9	1.087	0.923–1.280	0.319			
LYMP(×10^9^/L)^a^	1.2 ± 0.5	1.4 ± 0.6	0.631	0.337–1.182	0.151			
CRP (mg/L) ^a^	16.1 ± 5.4	19.7 ± 7.6	1.011	0.968–1.056	0.617			
RBC (× 10^12^/L)^a^	3.8 ± 0.5	3.9 ± 0.6	0.720	0.402–1.290	0.270			
Hb(g/L)^a^	116.3 ± 17.6	119.8 ± 19.4	0.990	0.973–1.007	0.263			
HCT(%)^a^	33.6 ± 4.9	36.7 ± 4.6	0.863	0.719–1.035	0.112			
PLT (×10^9^/L)^a^	206.7 ± 90.4	224.6 ± 94.6	0.998	0.994–1.002	0.247			
PT(s)^a^	11.5 ± 1.7	11.3 ± 1.6	1.089	0.891–1.330	0.406			
APTT(s)^a^	28.2 ± 4.7	27.4 ± 4.9	1.032	0.964–1.104	0.371			
TT(s)^a^	19.8 ± 2.9	19.4 ± 2.5	1.062	0.937–1.203	0.346			
INR^a^	0.9 ± 0.1	0.9 ± 0.2	1.763	0.180–7.228	0.626			
ALT (U/L) ^a^	112.3 ± 95.2	162.3 ± 78.8	0.997	0.995–1.000	0.057			
AST (U/L) ^a^	96.0 ± 65.1	115.6 ± 94.7	0.997	0.993–1.001	0.165			
ALP (U/L) ^a^	509.3 ± 390.1	498.4 ± 374.0	1.000	0.999–1.001	0.861			
TBIL ≥171.1 (μmol/L)								
No	34	76	1					
Yes	27	26	2.214	1.117–4.390	0.023	0.997	0.267–3.720	0.997
TP (g/L) ^a^	59.4 ± 2.8	61.7 ± 6.0	0.905	0.755–1.084	0.279			
Preoperative ALB ≥35.0 (g/L)								
No	25	26	1					
Yes	36	76	0.480	0.241–0.955	0.037	0.447	0.210–0.952	0.037
GLB (g/L) ^a^	25.7 ± 3.5	24.9 ± 3.7	1.062	0.853–1.323	0.588			
PA (mg/L) ^a^	201.8 ± 95.9	187.2 ± 71.7	1.002	0.997–1.007	0.366			
AMY (U/L) ^a^	85.0 ± 59.2	145.6 ± 106.3	0.996	0.988–1.004	0.347			
CA19–9 (U/mL)^a^	648.5 ± 133.7	622.8 ± 147.2	1.000	1.000–1.000	0.941			
Site of Lesion								
Distal common bile duct	18	17(16.7)	1		0.262			
Duodenal papilla	17(27.9)	32(31.4)	0.472	0.193–1.155	0.100			
Pancreatic head	21(34.4)	41(40.2)	0.561	0.234–1.342	0.194			
Ampullary	5(8.2)	12(11.8)	0.747	0.557–1.003	0.052			
***Intra-Operative factors***								
Operation time (h) ^a^	7.6 ± 1.5	7.8 ± 1.7	0.923	0.754–1.129	0.435			
Estimated Blood loss(ml) ^a^	591.5 ± 451.2	497.4 ± 311.1	1.001	1.000–1.002	0.128			
pRBC transfusion (U) ^a^	1.7 ± 0.6	1.5 ± 0.5	1.081	0.881–1.325	0.455			
Plasma transfusion(ml)	237.5 ± 80.3	132.8 ± 50.9	1.002	1.000–1.004	0.009	1.002	1.000–1.003	0.057
Total liquid volume(ml)	5829.2 ± 1431.7	6171.3 ± 1544.2	1.000	1.000–1.000	0.275			
Ratio of crystal fluid/colloid fluid	1.8 ± 0.5	2.3 ± 0.6	0.219	0.046–1.040	0.056			
Pancreatic gland texture								
Soft	36	58	1					
Hard	25	44	0.603	0.322–1.129	0.114			
Pancreatic duct diameter > 0.3 cm								
No	27	25	1			1		
Yes	34	77	0.425	0.215–0.839	0.014	0.819	0.369–1.815	0.622
PJ-R Type								
IPJ (external stenting)	49	81	1		0.219			
DMPJ (external stenting)	0	2	–	–	–			
DMPJ (internal stenting)	4	14	1.189	0.449–3.152	0.728			
IPJ (no stenting)	5	4	5.833	0.953–7.717	0.056			
IPJ (internal stenting)	3	1	1.460	0.914–2.330	0.113			
Combined portal vein resection								
No	60	100	1					
Yes	1	2	1.180	0.105–3.296	0.893			
Resectional margin status								
R0^b^	53	91	1		0.697			
R1	5	5	1.692	0.468–6.124	0.423			
R2	3	6	1.046	0.559–1.956	0.889			
***Pathological characteristics and postoperative complications***								
Pathology								
Adenocarcinoma	59(96.7)	85(83.3)	1		0.949			
Mucinous adenocarcinoma	0(0)	5(4.9)	–	–	–			
Inflammation mass	2	10	0.754	0.565–1.005	0.054			
Sarcoma	0	1	–	–	–			
Retention cyst	0	1	–	–	–			
Tumor Differentiation								
Inflammation	2	11	1		0.570			
High	26	45	0.900	0.356–2.274	0.824			
Moderate	23	27	1.033	0.592–3.974	0.379			
Poor	10	19	1.167	0.758–1.797	0.484			
Clavien-Dindo grade								
0	28	61	1		0.262	1		0.173
I	11	25	0.472	0.193–1.155	0.100	0.620	0.105–3.656	0.597
II	12	6	0.561	0.234–1.342	0.194	1.127	0.179—7.085	0.899
IIIa	2	5	3.765	0.897–9.296	0.067	1.582	0.316–7.927	0.577
IIIb	2	4	3.351	1.641–6.845	0.001	4.317	1.479–12.602	0.007
IVa	2	0	–	–	–	–	–	–
IVb	0	0	–	–	–	–	–	–
V	4	1	1.654	1.186–2.306	0.003	1.605	1.097–2.349	0.015

*Abbreviations: OR odds ratio, BMI* body mass index, *TBIL* total bilirubin, *ALT* alanine aminotransferase, *AST* aspartate aminotransferase, *ALP* alkaline phosphatase, *TP* total protein, *GLB* globulin, *ALB* albumin, *PA* prealbumin, *AMY* amylase, *CRP* C-reactive protein, *RBC* red blood cell count, *WBC* white blood cell count, *NEUT* neutrophil count, *LYMP* lymphocyte count, *Hb* hemoglobin, *PLT* platelet count, *HCT* hematocrit, *PT* prothrombin time, *APTT* activated partial thromboplastin time, *TT* thrombin time, *INR* international normalized ratio, *CA19–9* carbohydrate antigen 19–9, *pRBC* packed red blood cell, *PJ-R* pancreaticojejunostomy reconstruction, *DMPJ* duct-to-mucosa end-to-side pancreaticojejunostomy, *IPJ* invagination pancreaticojejunostomy

^a^ expressed as Mean ± SD;

^b^The resectional margin status in non-malignant disease was classified and analyzed as R0

### Postoperative complications

A total of 89 (54.6%) cases had no complications. According to classification based on the Clavien– Dindo criteria, the percentages of all patients who experienced grade I to grade V complications were grade I 22.1% (36/163), grade II 11.0% (18/163), grade IIIa 4.3% (7/163), grade IIIb 3.7% (6/163), grade IVa 1.2% (2/163), grade IVb 0% (0/163), and grade V 3.1% (5/163), respectively. The 30-day in-hospital mortality was two cases in HA group and one case in non-HA group, and 60-day in-hospital mortality was three cases in HA group and two cases in non-HA group, showing no significant difference.

The most common complication was ascites (37.4%), followed by infection (30.1%), pancreatic fistula (18.4%), and postoperative upper gastrointestinal tract hemorrhage (6.1%). Five patients presented with early intraperitoneal hemorrhage, of which two cases underwent reoperation and three received conservative treatment, two cases were cured after conservative treatment, and one died due to liver and kidney dysfunction. Nine cases experienced late intraperitoneal hemorrhage, of which three cases received laparotomy after hemostatic failure, two cases underwent interventional embolization treatment, and four cases were treated with double-catheter irrigation and local application of hemostatic drugs.

The incidence of upper gastrointestinal hemorrhage was 6.1% (10/163), including one case that experienced early upper gastrointestinal bleeding within 12 h and was treated with hemostatic drugs and nine cases with late bleeding within 3 ~ 23 days postoperatively that resolved after conservative treatment. Five patients presented with biliary fistula (3.1%), including one case of both pancreatic fistula and late intraperitoneal hemorrhage that was treated by reoperation. The percentages of patients who experienced DGE of grades B and C were 1.8% (3/163) and 1.2% (2/163), respectively, and hyperbilirubinemia affected 6.13% (10/163) of the patients.

Most complications were evaluated as grade I, including incision site infection and urinary tract infection. Intra-abdominal abscess formation (1.8%) was the most common type of infection among cases with grade II complications.

### Factors influencing HA after PD

Univariate analysis showed that the following five factors were associated with postoperative HA: preoperative TBIL ≥171.1 μmol/L, preoperative ALB ≥35.0 g/L, classification of complications, main pancreatic duct inner diameter > 0.3 cm, and intraoperative plasma transfusion (Table 1). Multivariate analysis showed that preoperative ALB ≥35.0 g/L and the complication grade were the factors significantly influencing HA after PD (Table 1).

### Correlations of preoperative ALB level and ALB infusion with various indicators

As shown in Table 2, liver function, inflammatory reaction index, coagulation, renal function and electrolyte index, postoperative complications, digestive tract anastomotic leakage, postoperative RBC and plasma transfusion, ascites, and diarrhea and somatostatin application exhibited correlation with ALB level and ALB infusion amount. No coagulation index was relevant with ALB level and ALB infusion amount. Surgical treatment of complication, early and late postoperative intraperitoneal hemorrhage were significantly correlated with HA. RBC and plasma transfusion may not affect serum ALB level. High request of RBC transfusion, such as severe complication, may generate the clinical circumstance of ALB infusion to correct HA. ALB level showed no correlation with ascites formation and volume. ALB infusion amount exhibited weak positive correlation with ascites, and weak negative correlation with positive germiculture in abdominal drainage. Somatostatin application and diarrhea after operation showed no correlation.

**Table 2:** Correlation analysis of postoperative ALB level, infused exogenous ALB and various indicators

Item	r	Wilk’s	χ^2^	DF	*P* value	U	V
Liver function	0.999	0.001	1815.225	26.000	0.000	0.414 × ALB level+ 1.082 × ALB infusion amount	−0.001 × ALT + 0.000 × AST + 0.003 × ALP – 1.697 × TP+ 1.160 × GLB − 0.006 × PA + 0.004 × TBIL
Blood routine	0.678	0.467	202.342	22.000	0.000	−0.652 × ALB level+ 0.547 × ALB infusion amount	− 0.385 × RBC + 0.176 × Hb – 0.137 × HCT–3.084 × WBC − 3.327 × NEUT–0.001 × LYMP − 0.475 × PLT–0.765 × CRP
Coagulation	0.531	0.598	63.715	14.000	0.000	1.044 × ALB level + 0.111 × ALB infusion amount	0.218 × PT + 0.093 × APTT + 0.091 × TT + 0.347 × INR
Renal function and electrolyte	0.997	0.002	37.224	18.000	0.005	0.487 × ALB level– 0.675 × ALB infusion amount	0.284 × Na^+^+ 0.056 × K^+^+ 0.248 × CL^−^–0.025 × Ca^2+^+ 0.220 × Mg^2+^ −0.296 × P– 1.737 × Urea–2.597 × Cr
Blood and drainage amylase and lipase	0.562	0.671	11.776	8.000	0.162	–	–
Clavien-Dindo Grade, PIH and reoperation	0.289	0.905	23.532	10.000	0.009	0.323 × ALB level + 1.092 × ALB infusion amount	−0.441 × Clavien-Dindo grade + 1.026 × reoperation + 1.298 × EPIH + 0.993 × LPIH
Anastomotic leakage	0.422	0.783	52.835	10.000	0.000	−0.408 × ALB level + 0.754 × ALB infusion amount	– 0.180 × biliary fistula+ 1.219 × PF + 0.540 × PF classification − 0.162 × infectious complications
Hyperbilirubinemia	0.125	0.984	3.611	2.000	0.164	–	–
Gastrointestinal hemorrhage	0.417	0.825	3.940	4.000	0.414	–	–
Postoperative pRBC and plasma transfusion	0.176	0.962	11.183	4.000	0.025	−0.527 × ALB level– 1.074 × ALB infusion amount	−0.801 × pRBCtransfusion − 0.372 × plasma transfusion
Ascites, pleural effusion and intraabdominal drainage germiculture	0.413	0.799	49.609	8.000	0.000	−0.122 × ALB level+ 0.944 × ALB infusion amount	0.575 × ascites + 0.372 × ascites severity+ 0.440 × pleural effusion − 0.651 × positive germiculture − 0.107 × positive fungus culture
Diarrhea, somatostatin analogs use	0.430	0.795	12.740	4.000	0.013	−0.311) × ALB level – 0.996 × ALB infusion amount	−0.441 × diarrhea − 0.002 × somatostatin analogs use

*Abbreviations: PIH* postoperative intraperitoneal hemorrhage, *EPIH* early postoperative intraperitoneal hemorrhage, *LPIH* late postoperative intraperitoneal hemorrhage, *PF* pancreatic fistula

### Comparison of postoperative parameters between HA and non-HA groups

No significant difference was observed in the rates of complications between the HA and non-HA groups (Table 3). Moreover, no differences were observed in the total amount of ALB infused (*P* = 0.186), ALB infusion frequency (*P* = 0.365), or postoperative 30, 60-day fatality rate (*P* = 0.557, 0.066, respectively) between the two groups. The classes of complications showed significant difference between the two groups (*P* = 0.020).

**Table 3:** Comparison of postoperative parameters between HA and non-HA groups

Parameters	HA(*n* = 61)	non-HA(*n* = 102)	*P* value ^*a*^
EPPH	3	2	0.360
EPPH treatment			1.000
Non-surgical therapy	2	1	
Reoperation	1	1	
LPPH	4	5	0.729
LPPH treatment			
Conservation	1	3	0.539
Intervention	1	1	
Surgery	2	1	
POPF	12	18	0.747
POPF grade			0.076^*b*^
A	6	14	
B	2	3	
C	4	1	
Biliary fistula	1	4	0.651
DGE Grade C	2	3	1.000
Hyperbilirubinemia	4	6	1.000
Infection complications	20	29	0.557 ^*c*^
Infection site			
Incision	15	15	0.079 ^*c*^
Lung	1	0	
Intra-abdominal	4	14	
Ascites	26	35	0.289^*c*^
Ascites severity			
Mild	14	25	0.157^*c*^
Middle	12	10	
Postoperative delirium	3	1	0.148
Cardiovascular events	1	0	0.374
Liver dysfunction	2	0	0.139
Upper gastrointestinal hemorrhage	5	5	0.503
Lower gastrointestinal hemorrhage	1	0	0.374
Multiorgan dysfunction	2	1	0.557
Clavien-Dindo classification			0.020^*b*^
0	28	61	
I	11	25	
II	12	6	
IIIa	2	5	
IIIb	2	4	
IVa	2	0	
IVb	0	0	
V	4	1	
Reoperation	2	4	1.000
Postoperative ALB infusion dosage(g) *	169.0 ± 126.1	115.6 ± 87.9	0.186 ^*d*^
Postoperative ALB infusion frequency*	10.7 ± 6.9	5.81 ± 4.0	0.365^*d*^
Somatostatin application (day) *	9.3 ± 4.2	10.1 ± 3.8	0.215 ^*d*^
Postoperative pRBC transfusion(U) *	4.3 ± 3.1	4.0 ± 1.9	0.003 ^*d*^
Postoperative plasma transfusion (ml) *	869.0 ± 633.8	545.9 ± 373.1	0.086 ^*d*^
Hospital stay (days) *	30.0 ± 13.6	29.2 ± 13.4	0.658 ^*d*^
30-day Mortality	2	1	0.557
60-day Mortality	4	1	0.066

*a: Fisher’s exact test; b: Wilcoxon rank sum test; c: Pearson x*^*2*^*test; d: Mann-Whitney U test*

*Abbreviations: EPPH* early postpancreatectomy hemorrhage, LPPH Late postpancreatectomy hemorrhage

* expressed as Mean ± SD

## Discussion

In this study, we analyzed the factors influencing HA after PD and the effects of HA on short-term prognosis. Multivariate analysis showed that preoperative ALB ≥35.0 g/L and the classification of postoperative complications significantly affected the occurrence of HA after PD, but the underlying mechanism is still elusive. We postulate that ALB level may indicate inflammation status in the body which may affect postoperative HA, but further studies are needed to confirm it.

Correlation analysis showed that postoperative ALB level was only negatively correlated with WBC and neutrophil counts. The amount of exogenous ALB infused postoperatively was negatively correlated with TP but positively correlated with BUN, Cr, complications requiring surgical intervention, early intraperitoneal hemorrhage, late intraperitoneal hemorrhage and pancreatic fistula. Importantly, the incidences of complications and the 30-day mortality rates did not differ significantly between HA group and non-HA group.

About 20–40% of patients present with HA after selected operations [20]. Exogenous ALB infusion may correct postoperative HA and maintain intravascular colloid osmotic pressure. However, the endothelial glycocalyx (EG) layer plays a role in intra- and extracellular liquid exchange, reabsorption of interstitial fluid may not occur even if exogenous ALB is infused to improve plasma colloid osmotic pressure [21]. In addition, although ALB is a key component of the EG layer, only 25% of physiological concentration of ALB can maintain the complete EG layer, and EG layer remains functional even with an ALB level of 10 g/L [22]. Thus, exogenous ALB infusion is not recommended to improve and maintain the function of the EG layer.

ALB level after PD and infusion of exogenous ALB exhibited no relationship with HCT in this study, indicating that hemodilution was not the main reason for HA, consistent with previous report [20]. The integrity and function of the EG layer suffer damage during inflammatory response [23]. Excessive inflammation leads to the release of many inflammatory mediators and increased number of blood leukocytes, especially neutrophils, contributing to EG layer barrier dysfunction, leukocyte adhesion to the vascular wall and increased vascular permeability [24].

This study revealed that postoperative ALB level negatively correlated with postoperative inflammation indexes, such as WBC count, neutrophil absolute value, and CRP level. The correlation of CRP with postoperative ALB level was lower than that of the WBC and neutrophil counts, suggesting that WBC and neutrophils, especially neutrophils, have specific roles in the ALB decline after PD, probably via damage to the EG layer. We speculated that the surgery itself, as the initial stimulus of inflammation, led to an increase in the WBC count, especially in neutrophils. The release, activation and dynamic changes of different subgroups of neutrophils contributed to the damage and subsequent dysfunction of the EG layer. This may explain why infusion of exogenous ALB cannot reliably improve HA in some patients and even correcting ALB level > 30 g/L does not effectively prevent serious complications. Furthermore, such damage and functional loss of the EG layer may not be resolved while inflammation subsides during recovery, as reflected by a decrease in the neutrophil count, because serum ALB level slowly returns to the normal range over a relatively long time after the operation.

Anastomotic fistula after PD, including bile leakage, pancreatic leakage, and intra-abdominal abscess formation, is the most serious complication that significantly increases postoperative mortality, and may become aggravated as late as the 8-12th day [25]. This study demonstrated that a reduction in postoperative ALB may not affect the occurrence of pancreatic fistula. Moreover, infusion of exogenous ALB could not prevent the occurrence of pancreatic fistula. However, exogenous ALB infusion was positively correlated with surgical treatment of postoperative complications and postoperative intraperitoneal hemorrhage, especially the early postoperative intraperitoneal hemorrhage. Early hemorrhage after PD can be caused by the operative technique or coagulation dysfunction, which usually requires abdominal laparotomy to stop the bleeding [1]. For the patients who underwent reoperation, two operations in a short period induced excessive inflammation, leading to ALB decrease. In this case, the clinician may use exogenous ALB to correct HA. However, late postoperative bleeding usually occurs in 1–3 weeks after surgery. The cause of this complication is complex and may be related to peripancreatic blood vessel corrosion secondary to pancreatic leakage, aneurysm formation, or ulceration of anastomotic site [26]. Generally, postoperative bleeding can be stopped upon interventional therapy or endoscopic treatment under local anesthesia. Our results suggest that postoperative RBC or plasma transfusion did not improve ALB level.

Based on our results, the strategy for correcting HA after PD is to block or rapidly end the acute phase of the inflammatory response and protect or restore the EG layer, although the clinical benefit of this approach is questionable. In addition, careful attention to perioperative nutritional support can help patients pass through the acute stress reaction period, even though parenteral nutrition has been shown to seldom correct ALB concentration to normal level [27].

There are several limitations in this study. First, PD procedures were performed by different physicians, and thus the amounts of fluid infused daily and the ratio of crystal and colloid varied. The impact of such differences on the occurrence of postoperative complications is difficult to evaluate. Second, exogenous ALB preparation used in this study was 20% concentration, and the impact of other concentrations of ALB on HA after PD remains unclear. Third, the variety of diagnoses may contribute to possible bias in our conclusion. Fourth, we only evaluated short-term outcomes of patients after PD and exogenous ALB infusion. Fifth, the sample size is not big enough. Further multi-center randomized prospective studies with long-term follow-up are needed.

## Conclusions

Our study provides further evidence that HA should not be considered as an indicator but rather a result of poor prognosis. WBC, especially neutrophils, exhibit unique role in reducing postoperative ALB level. Infusion of exogenous ALB to maintain ALB > 30 g/L has no clinical benefit for patients after PD.

## Data Availability

All data used in the study are available from correspondence author Bo Jiang upon reasonable request.

## Abbreviations

ALB: Albumin
HA: Hypoalbuminemia
PD: Pancreaticoduodenectomy
